# Creating Low-Cost 360-Degree Virtual Reality Videos for Hospitals: A Technical Paper on the Dos and Don’ts

**DOI:** 10.2196/jmir.9596

**Published:** 2018-07-16

**Authors:** Benjamin O’Sullivan, Fahad Alam, Clyde Matava

**Affiliations:** ^1^ Department of Anesthesia and Pain Medicine Hospital for Sick Children, Toronto Toronto, ON Canada; ^2^ Department of Anesthesia University of Toronto Toronto, ON Canada; ^3^ Department of Anesthesia Sunnybrook Health Sciences Centre Toronto, ON Canada

**Keywords:** 360-degree video, VR, virtual reality, video production, anesthetic preparation, preoperative anxiety, preoperative preparation

## Abstract

This article will provide a framework for producing immersive 360-degree videos for pediatric and adult patients in hospitals. This information may be useful to hospitals across the globe who may wish to produce similar videos for their patients. Advancements in immersive 360-degree technologies have allowed us to produce our own “virtual experience” where our children can prepare for anesthesia by “experiencing” all the sights and sounds of receiving and recovering from an anesthetic. We have shown that health care professionals, children, and their parents find this form of preparation valid, acceptable and fun. Perhaps more importantly, children and parents have self-reported that undertaking our virtual experience has led to a reduction in their anxiety when they go to the operating room. We provide definitions, and technical aspects to assist other health care professionals in the development of low-cost 360-degree videos.

## Introduction

This article aims to provide both methods and practical advice for the production of immersive 360-degree videos for children in hospitals. It is targeted at those with previous experience in the production of standard videos. Preoperative preparation of children is a well-researched method of reducing perioperative anxiety and the consequences of this anxiety [1]. The advancements in immersive 360-degree technologies have allowed for producing a “virtual experience” where children can prepare for anesthesia by actually “experiencing” all the sights and sounds of receiving and recovering from an anesthetic.

There are many standard videos available online aimed at the preparation of children for their hospital procedures. It would also be possible to produce these videos in panoramic or 180-degree modes, which would be technically much easier to produce. However, children and their parents prefer using a 360-degree video. The benefits of 360-degree video over these other methods are threefold [2,3]:

1. It provides a full visual account of what the child could see, there is less chance of surprise at the time of their anesthetic.
2. The increased autonomy for the child during the process of preparation may itself lead to reduced levels of anxiety on the lead up to their anesthetic.
3. The use of virtual reality (VR) headsets or 360 video viewers, by the very nature of being a toy, may reduce anxiety by the child associating the anesthetic with something fun.

Furthermore, it has been demonstrated that health care professionals, children, and their parents find this form of preparation valid, acceptable, and fun [2,3]. Perhaps more importantly, children, and parents have self-reported that undertaking our virtual experience has led to a reduction in their anxiety when they go to the operating room (OR) [2,3]. We provide definitions and technical advice to assist other health care professionals in the development of low-cost 360-degree videos.

The production of 360-degree videos is significantly more challenging than the production of standard videos. There are many production steps which should be planned: (1) production of a script and recruitment of actors, (2) filming individual 360-degree scenes with an appropriate camera, (3) footage from each camera needs to be added together to produce the completed 360-degree video during a process called stitching, (4) the 360-degree footage needs to be edited into the completed film with appropriate software, and (5) the video file needs to be loaded onto a device supporting 360-degree video with a 360-degree viewer.

## Production of the Script and Recruitment of Actors

### Content and Pace of Video

As with planning the production of any video, it is essential to carefully consider what information the child will gain from the experience. It is important to optimize the length and pace of the video to ensure that it contains all the necessary information and does not result in symptoms such as motion sickness, dizziness, and headaches. An unnecessarily long video may increase the incidence of such symptoms or boredom, and the pace of the video should mimic real anesthetic experience as much as possible.

A key element in this is to perform a needs assessment to gain perspective from all stakeholders. This includes surveys, interviews, and a review of pre-existing standard videos created. Decision points for 360-degree videos will include deciding which elements of standard videos will work in that format.

Textbox 1 shows the basic structure of the video which lasts approximately 6 minutes. This serves as an example of using a needs assessment to inform the critical steps of the education 360 experience created for the viewer. The video was aiming to alleviate the anxiety of the preoperative experience. Textbox 1 has the 6 important phases that educate the viewer about this process. From a survey of 300 people (ie, 100 health care professionals, 100 parents, and 100 children), 291/300 (97%) rated the amount of information as optimal, and 288/300 (96%) rated the pace as optimal. Of particular note, none of the children desired any additional information nor would have liked any information to be deleted [2,3,4].

### Actors

The virtual experience appears more realistic if it flows naturally through the individual scenes rather than shorter sequences being pasted together. For this reason, it is much easier to use professional actors or staff that are acting in their usual professional roles. Even the most experienced staff member may need several takes to get the scene completed perfectly when the camera is rolling.

**The structure of the virtual experience.**

1. The preoperative area: Introduction and orientation from the nurse.
2. Walking down the corridor: The nurse explains where they are going and what they will see.
3. Walking into the OR: Meeting everyone in the OR and being asked to get onto the bed.
4. Lying on the operating bed: The nurse explains what she is doing while attaching all the routine monitors.
5. The anesthetist explains what they are doing while delivering a gas induction or inserting an intravenous (IV) tube and delivering an IV induction. The screen fades to black depicting falling asleep.
6. A brief period of darkness indicating being asleep.
7. In postoperative care unit: The nurse explains that procedure has finished, points out remaining pieces of monitoring, IV access, and reassures the child that the parents will be arriving soon.

## How to Film a 360-Degree Video

### Choosing a 360-Degree Camera

There exists an increasing number of 360-degree cameras available to the consumer. They all have different technical specifications, associated software, and workflows for stitching images. These vary in cost as well as using rigged or non-rigged cameras. A framework and rationale for the cameras selected is provided.

### Rigged Cameras or Dual Lens Cameras

**Figure 1:**
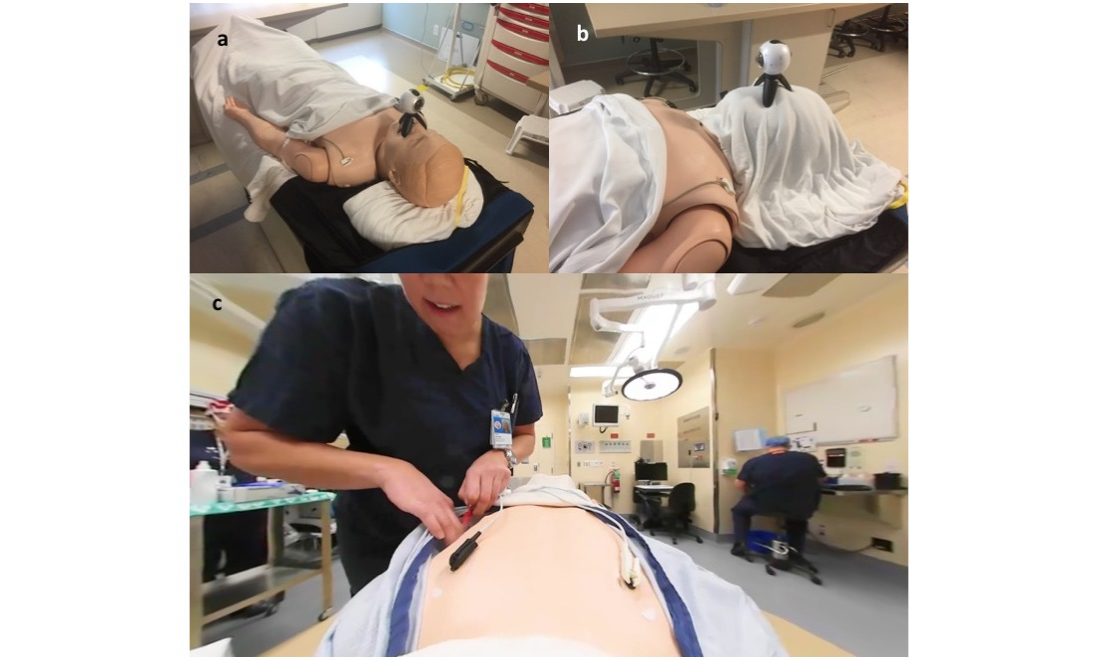
a) Optimal setup position of 360 camera; b) Optimal setup position of 360 camera with sheet covering the face; c) View through 360-degree viewer.

Two different set ups of cameras for shooting 360-degree videos were tested: (1) GoPro rig and (2) Ricoh Theta S. The first comprises a frame with 6 cameras fixed to it pointing along all 6 axes. The second was a self-contained system with two 180-degree lens cameras located back to back. Although the GoPro rigged system was superior concerning video quality, 3 significant disadvantages of this system over the self-contained 2 camera system were found. First, it was significantly bulkier and heavier which impeded setting up the camera in a number of the scenes. Second, there is footage from 6 cameras that need stitching together as opposed to 2 (dual lens camera), greatly increasing the postproduction work-load due to the increased amount of space between cameras and the amount of footage not captured, this set-up became inferior when filming objects at close range, such as face masks (Figure 1).

### Camera Definition

Resolution of video footage can be considered as the amount of information the camera collects in each frame. It is essential to understand that to produce a clear picture in immersive videos, a higher definition is required. This is for two main reasons. First, each frame of the video is not being watched in its entirety on a flat screen in front of the user. The information in each frame is, in fact, being stretched around a central point into a full 360-degree environment leading to a significant loss of resolution (Multimedia Appendix 1 and Multimedia Appendix 2). Second, the video is viewed at a distance of about 2 inches from the user’s eyes, within the 360-video viewer, and therefore any reduction in definition becomes magnified.

The first video was filmed using the Ricoh Theta S which claims a high-resolution of 1920 by 1080. Although the auto-stitch feature, which works well, the end product when viewed in the 360-degree viewer was of standard definition. 3/100the video complained of a blurry picture, sore eyes, and a headache. This was attributed to the low resolution of the video [3].

For the second video, the Samsung Gear 360 was used which has a higher video resolution of 3840 by 1920. Although this device does not come with an auto-stitch feature, it can produce a much clearer higher definition immersive experience. Although not formally assessed, the higher resolution appears to cause less incidence of sore eyes and headaches.

### How to Obtain a 360-Degree Footage

When producing non 360-degree films, the footage can be viewed as it is filmed. With 360-degree filming, everything in the line of sight of the camera is recorded, and it is not until after the stitching and into the editing process that the scene can be viewed entirely. It is a worthwhile exercise to have dry runs of each scene with someone in the position of the camera looking around the entire room to see exactly what the camera will see during the filming. This is especially important when filming in a hospital environment as it is easy to inadvertently film sensitive material such as a patient in the background or a piece of patient identifiable data on a computer screen.

The optimal way to film the experience was to use the camera as the head of the “patient” and administer an anesthetic to the camera. This ensured that the scene felt real and also enabled the user to feel like they are being spoken to and interacted with personally during their virtual experience.

To film the torso of the child and depict the application of various physiological monitors and lines, it is possible to either use a mannequin or an actor. The method we describe here uses a mannequin, but the same principles may be applied to a live model.

The camera needs to be positioned on the mannequin so that the child sees the “body” as they would their own. After testing different set-up options, the optimal position to set up the camera is as depicted in Figure 1a and b. This enables the child to see everything that happens during the induction of anesthesia and the application of monitoring equipment as shown in Figure 1c.

**Figure 2:**
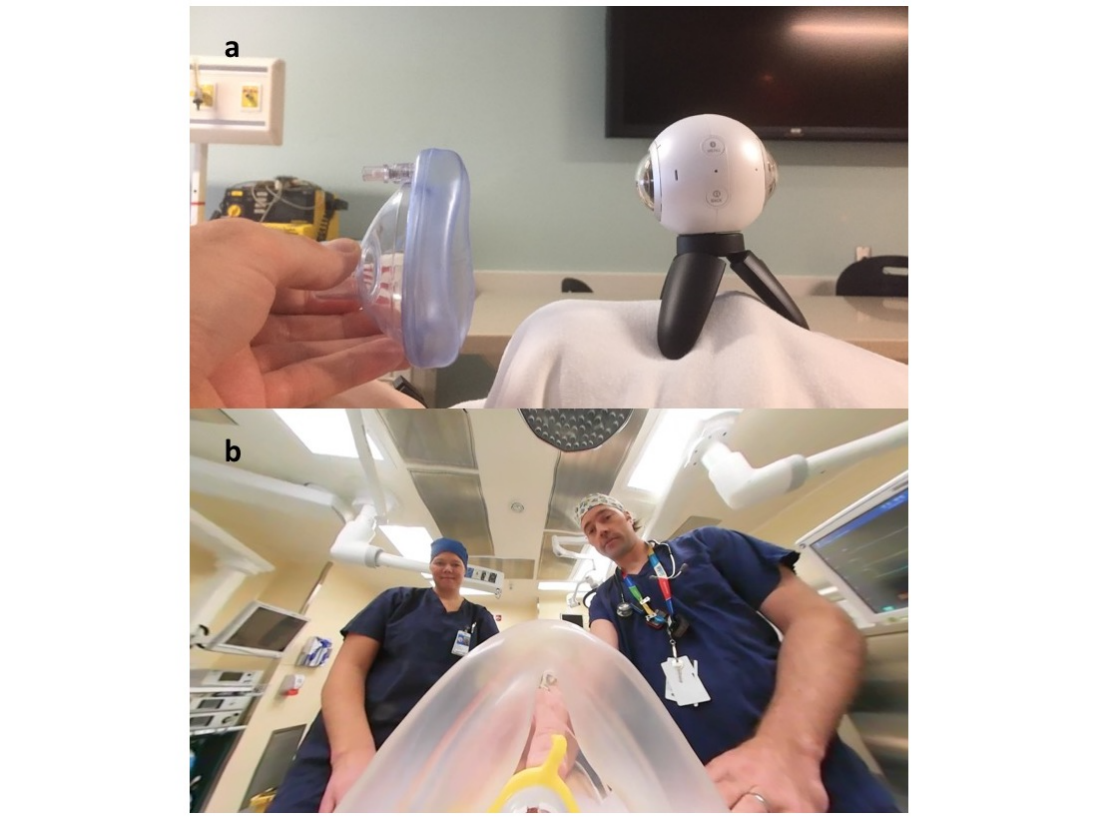
a) Optimum position of the mask during a gas induction; b) View of this setup through the 360-degree viewer.

One aspect of preanesthetic preparation for children which the virtual experience lends itself particularly well to is the gas induction. Holding the top of the mask 3-4 inches away from the camera and 1 inch above the center of the camera allows the child to experience how the mask will appear during a gas induction, as shown in Figure 2a. This allows the child to get the claustrophobic impression of the mask but also allows them still to see everything that is going on in the room. This is shown in Figure 2b. Children who have used the virtual experience have found this aspect of the preparation particularly useful.

**Figure 3:**
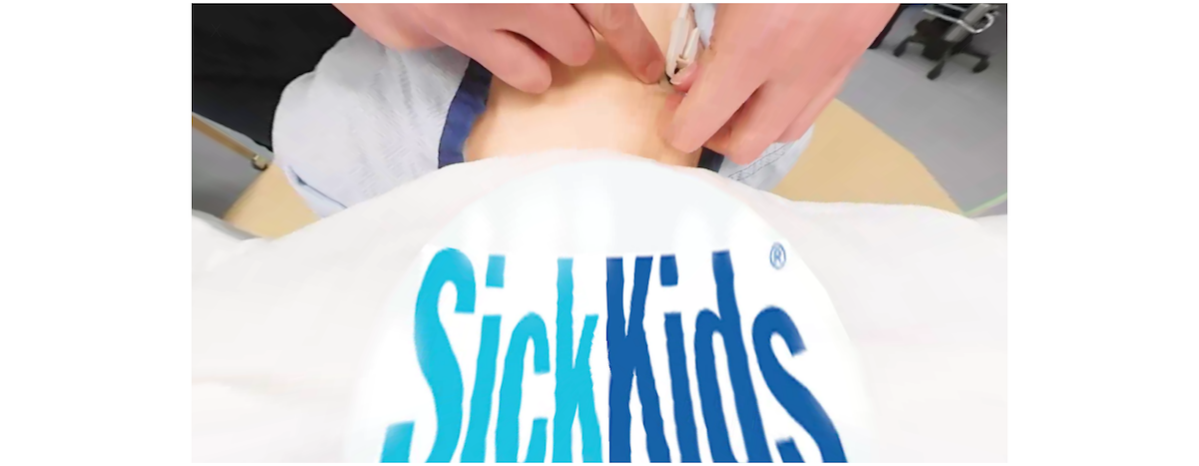
View through 360-degree viewer when looking down at our hospital’s logo.

One inherent technical problem was discovered when filming in this manner. Using the camera as the head of a body means that when the user looks down during the video, they will either see a face or a “headless” body depending on whether or not the face is covered. After multiple tests, it was discovered the most aesthetically pleasing method was to do 2 things. First, during the filming process, cover the face of the mannequin in a sheet the same color as the bed, as shown in Figure 1b. Second, during the editing process, place a logo at the bottom of the video to hide the space where the head would be located (Figure 3).

The scene depicting the walk to the OR posed another technical difficulty. In the first attempt, the nurse escorting the patient to the OR pushed an IV stand with the camera attached to the top. This initially appeared to work well; however, it did reveal one of the side effects caused by this technology. During the virtual experience, the user remains in a sitting position and is placed in a “virtual environment” where they perceive motion, which can lead to motion sickness. The first video caused 20/57 (35%) of users to feel dizzy and 10/57 (17.5%) to feel nauseous. Subjects attributed their side effects predominantly to this scene. The scene was refilmed using an IV stand with added weights at the bottom, to increase the stability of the pole, and wheeled it down the corridor much more slowly at a speed of about 0.25meters/second. This enabled the scene to be produced where the user still gets the impression of walking down the corridor but has a much lower incidence of motion sickness. The downside to this is that it was only possible to film a small portion of the walk to the OR as it would have taken too long to walk at this speed.

### Obtaining Audio for a 360-Degree Video

There are multiple ways of recording the audio for the video. The simplest option is to use the built-in microphone in the camera. This, however, does not provide the best audio quality, especially in an environment with lots of ambient noise. Voices from different locations within the environment will also be recorded at different volumes.

The most reliable way to record the actors’ voices is to fit each with microphones and add this to the video at the editing stage. Regarding background sounds, which are important for the experience, such as monitoring sounds or trays being opened, it is best to film the experience in a quiet room. Separately record these sounds and then add in during the editing. It is also possible to record actors’ voices at this stage.

### Stitching the Video Footage

This is a very time consuming and technically challenging task. Each frame from each camera needs to be stitched together to produce a video file that contains a full 360-degrees worth of information. The Ricoh Theta S camera allowed for a bypass to this stage as it had an auto stitch function enabling the export of data directly from the camera to the editing software. The Samsung Gear 360 did not come with this function, and it was necessary to obtain help from an outside company to complete the stitching process. This is a very significant consideration when deciding on which camera to use, as it may cost significantly more to hire outside help to complete the stitching of the footage.

## Editing and Combining Footage Into a Full Video

The editing techniques behind producing a completed video are beyond the scope of this article, and there are many editing software packages available. However, some of the fundamental concepts when editing a 360-degree video are explained here. The editing software used was Adobe Premiere.

### Equirectangular Videos

**Figure 4:**
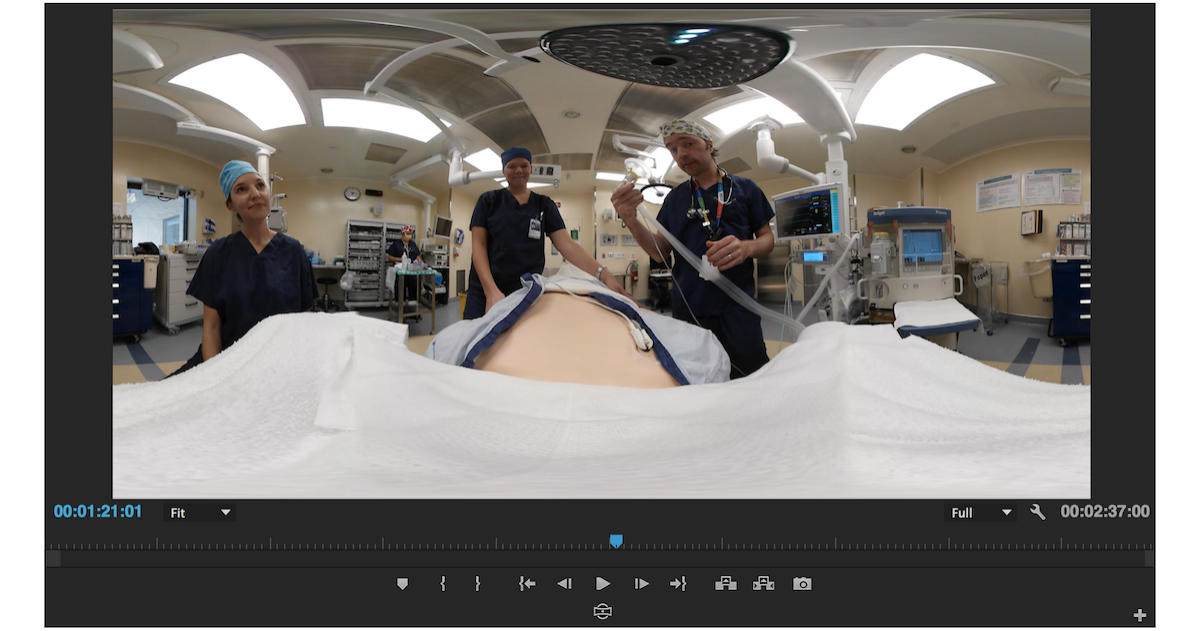
How 360-degree footage appears when flattened onto a 2-Dimensional workspace.

After the stitching process has been completed, the video files are in equirectangular format. This means that the 360-degree spherical images have been flattened and distorted onto a 2-dimensional rectangle, much like a map of the world. Therefore, when viewing the clips during the editing process, they will appear distorted, and it is difficult to appreciate what the final product will look like (Figure 4).

### Video Clip Settings

For the final product to work as required in the 360-degree video viewer, it is vital to maintain the correct relative dimensions of the video during the import process into the editing software and during the export process out of the editing software. The exact magnitude of the dimensions will vary depending on the resolution of the clips but will need to be twice as wide as they are high (ie, 3840 by 1920). It is also vital to ensure that there are no borders to the image as the edges of the video will be wrapped around and brought together when playing on the 360-degree video viewer.

### Lighting

Even though additional lighting was used during the filming, the finished product was still quite dark. Increasing the exposure setting by approximately 40% and the saturations setting by approximately 80% on the editing software produced a video that is much brighter and warmer. It also gives a much clearer picture. The exact degree to which these settings should be adjusted will depend on the original footage.

### Exporting Video From the Editing Software and Injecting Metadata

Table 1 shows the export settings that were used. The VR mode required will depend on the device used to view the final product. Most 360-degree editing software will automatically format the video file into a stereoscopic mode (ie, 2 images side by side) which is required to view the video file in the viewer. If software is used that does not do this, then it will be necessary to export as a stereoscopic video file.

### Metadata

Metadata is the information that is embedded into the video file that allows the video to be viewed in 360-degree mode. Whether or not the raw footage contains this data depends on the camera used. It may be necessary to install this metadata during the export process. Using this method, it is not necessary to do this. However, it is critical to ensure that when the video is exported from the editor that the metadata remains within the video file by enabling the metadata settings.

**Table 1:** Video export settings.

Attribute	Setting
Format	H.264
Width	3840
Height	1920
Frame Rate	29.97
Aspect	Square Pixels
Bitrate	Maximum Bitrate
Virtual reality mode	Monoscopic
Metadata	Enabled

## The 360-Degree Enabled Devices and Viewers

There are a large number of devices and viewers (eg, HTC Vive, Samsung Gear, Google Cardboard), that allow viewing 360-degree video media. The most reliable and cost-effective way of viewing the completed video is as follows. First, download the completed video to a mobile phone. All current mobile phones have the ability to run the necessary VR applications. Second, open the video in an appropriate VR application. The free and open source VR application called Childlife VR was used. Third, view the video through a 360 viewer. These are readily available devices for mobile phones. The Google Cardboard viewer was used in this study due to its availability and price.

For safety reasons, it must be ensured that all users of the video remain seated at all times during the experience. This still enables them to look all around and experience the full benefit of the video. Although most of the information happens in the “looking forward” position, children still choose to take advantage of the technology and look all around them throughout the video.

## Discussion

This tutorial provides an account of the technical challenges encountered and techniques that were found to be effective when producing a video (ie, Childlife VR). There are many different ways of preparing children for a hospital procedure and not all will wish to participate in this particular way. There was 1/ 101 (1%) of the children approached to use the video chose not to try it as they had received over 8 previous anesthetics. They had their own coping mechanism and did not want this process interfered with. In addition, 6/100 (6%) children recruited preferred to use the standard methods of preparation in the future [1,2].

Three-dimensional cameras and augmented reality goggles are becoming more readily available and more reasonably priced. This may represent future alternative methods used in the preparation of children for general anesthesia and other hospital procedures.

## Appendix

Multimedia Appendix 1 Comparison of rigged set up (left) with dual camera (right). (A) shows the areas not captured by the cameras; (B) shows the areas that need “stitching“ together

Multimedia Appendix 2 How footage is “stretched“ around the user in order to be viewed in 360-degrees.

## Abbreviations

IV: intravenous
OR: operating room
VR: virtual reality
