# Supplementary figures and images for: Creating Low-Cost 360-Degree Virtual Reality Videos for Hospitals: A Technical Paper on the Dos and Don’ts
Source: J Med Internet Res. 2018 Jul 16;20(7):e239. doi: 10.2196/jmir.9596 (PMC6066637; doi:10.2196/jmir.9596)

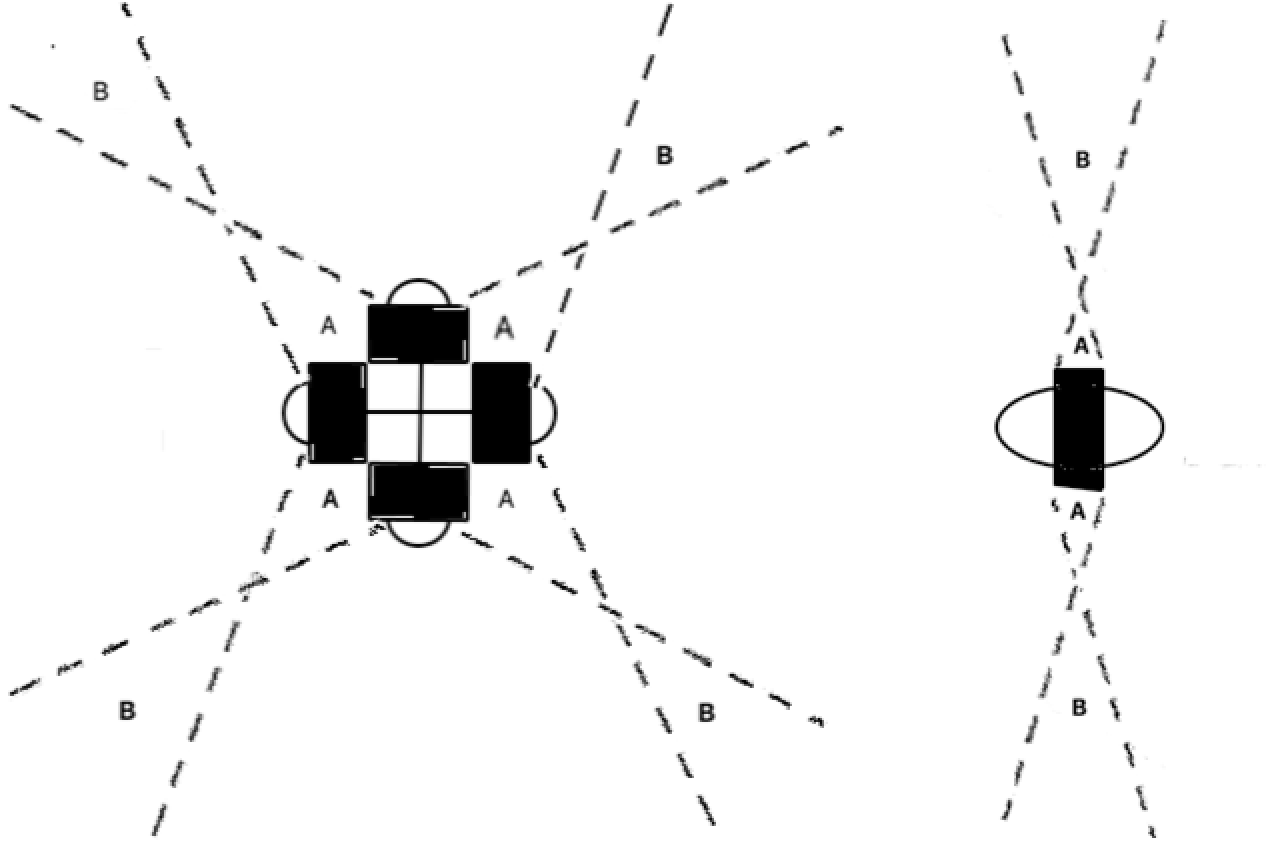

Supplement: Multimedia Appendix 1 [file jmir_v20i7e239_app1.png]

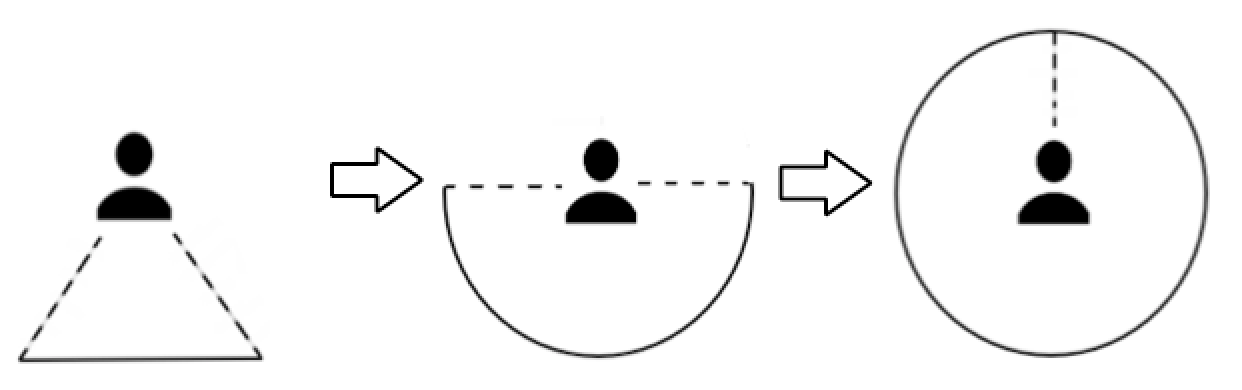

Supplement: Multimedia Appendix 2 [file jmir_v20i7e239_app2.png]
